# Resection of the mesopancreas (RMP): a new surgical classification of a known anatomical space

**DOI:** 10.1186/1477-7819-5-44

**Published:** 2007-04-25

**Authors:** Ines Gockel, Mario Domeyer, Tanja Wolloscheck, Moritz A Konerding, Theodor Junginger

**Affiliations:** 1 Department of General and Abdominal Surgery, Johannes Gutenberg-University of Mainz, Germany; 2 Institute of Anatomy and Cell Biology, Johannes Gutenberg-University of Mainz, Germany

## Abstract

**Background:**

Prognosis after surgical therapy for pancreatic cancer is poor and has been attributed to early lymph node involvement as well as to a strong tendency of cancer cells to infiltrate into the retropancreatic tissue and to spread along the peripancreatic neural plexuses. The objective of our study was to classify the anatomical-surgical layer of the mesopancreas and to describe the surgical principles relevant for resection of the mesopancreas (RMP). Immunohistochemical investigation of the mesopancreatic-perineural lymphogenic structures was carried out with the purpose of identifying possible routes of metastatic spread.

**Methods:**

Resection of the mesopancreas (RMP) was performed in fresh corpses. Pancreas and mesopancreas were separated from each other and the mesopancreas was immunohistochemically investigated.

**Results:**

The mesopancreas strains itself dorsally of the mesenteric vessels as a whitish-firm, fatty tissue-like layer. Macroscopically, in the dissected *en-bloc *specimens of pancreas and mesopancreas nerve plexuses were found running from the dorsal site of the pancreatic head to the mesopancreas to establish a perineural plane. Immunohistochemical examinations revealed the lymphatic vessels localized in direct vicinity of the neuronal plexuses between pancreas and mesopancreas.

**Conclusion:**

The mesopancreas as a perineural lymphatic layer located dorsally to the pancreas and reaching beyond the mesenteric vessels has not been classified in the anatomical or surgical literature before. The aim to ensure the greatest possible distance from the retropancreatic lymphatic tissue which drains the carcinomatous focus can be achieved in patients with pancreatic cancer only by complete resection of the mesopancreas (RMP).

## Background

The poor prognosis after surgical therapy for pancreatic cancer has been attributed to early lymph node involvement as well as to a strong tendency of the cancer cells to infiltrate into the retropancreatic tissue and to spread along the peripancreatic neural plexuses.

The pancreas is covered dorsally by a perineural layer, the mesopancreas. This is a firm and well-vascularized structure extending from the posterior surface of the pancreatic head to behind the mesenteric vessels (Superior Mesenteric Vein (SMV) and Superior Mesenteric Artery (SMA)). The course of lymphogenic structures along the neuronal plexus posteriorly to the pancreas may have a key role in metastatic spread. Perineural tumor invasion has been detected in up to 77% of the resection specimens from patients with carcinoma of the head of the pancreas [1,2].

The crucial importance of the surgical principle of the "holy plane" in rectal carcinoma was described by Heald in 1982 [3]. The introduction of the total mesorectal excision (TME) has lead to a significant decrease in the loco-regional occurrence rate and thus to an improvement in the long-term prognosis for carcinoma of the rectum.

An analysis of the available literature did not yield a corresponding definition of the mesopancreas or any data on the surgical resection of the structure as an intact entity comprising the pancreas and the mesopancreas.

The aim of this study was therefore both the anatomical-surgical classification of this layer on the basis of resection specimens obtained from fresh corpses. In addition, an immunohistochemical investigation of the mesopancreatic-perineural lymphogenic structures was carried out with the purpose of identifying possible routes of metastatic spread.

Embryologically, the parenchyma of the pancreas develops from a ventral and a dorsal endodermal bud arising from the later duodenum. The *dorsal *pancreas bud, which unfolds and extends into the dorsal "mesoduodenum" above and adjacent to the liver bud, is of greater importance. The epithelial bud arises here dorsally to the stomach and unfolds in a left lateral direction. As the bud migrates dorsally, the ventral pancreas inclusive of the region around the outlet of the common bile duct fuses with the dorsal pancreas from caudal. Based on the findings of histological investigations in human embryos, Borghi *et al*., demonstrated that the complete fusion of the two pancreatic buds is achieved considerably later than has previously been assumed [4]. The authors found a close ontogenetic relationship between the dorsal pancreas and the lymphatic and neuronal structures in the dorsal mesogastrium, which later forms the retropancreatic connective tissue, while the relationship with other lymphatic structures was confined to the ventral pancreas bud [4].

The pancreas is supplied with postganglionic sympathetic and parasympathetic innervation. Coursing parallel to these efferent nerve fibers, a large number of afferent viscerosensitive nerve fibers arising from the pancreas proceed centrally.

**Figure 1:**
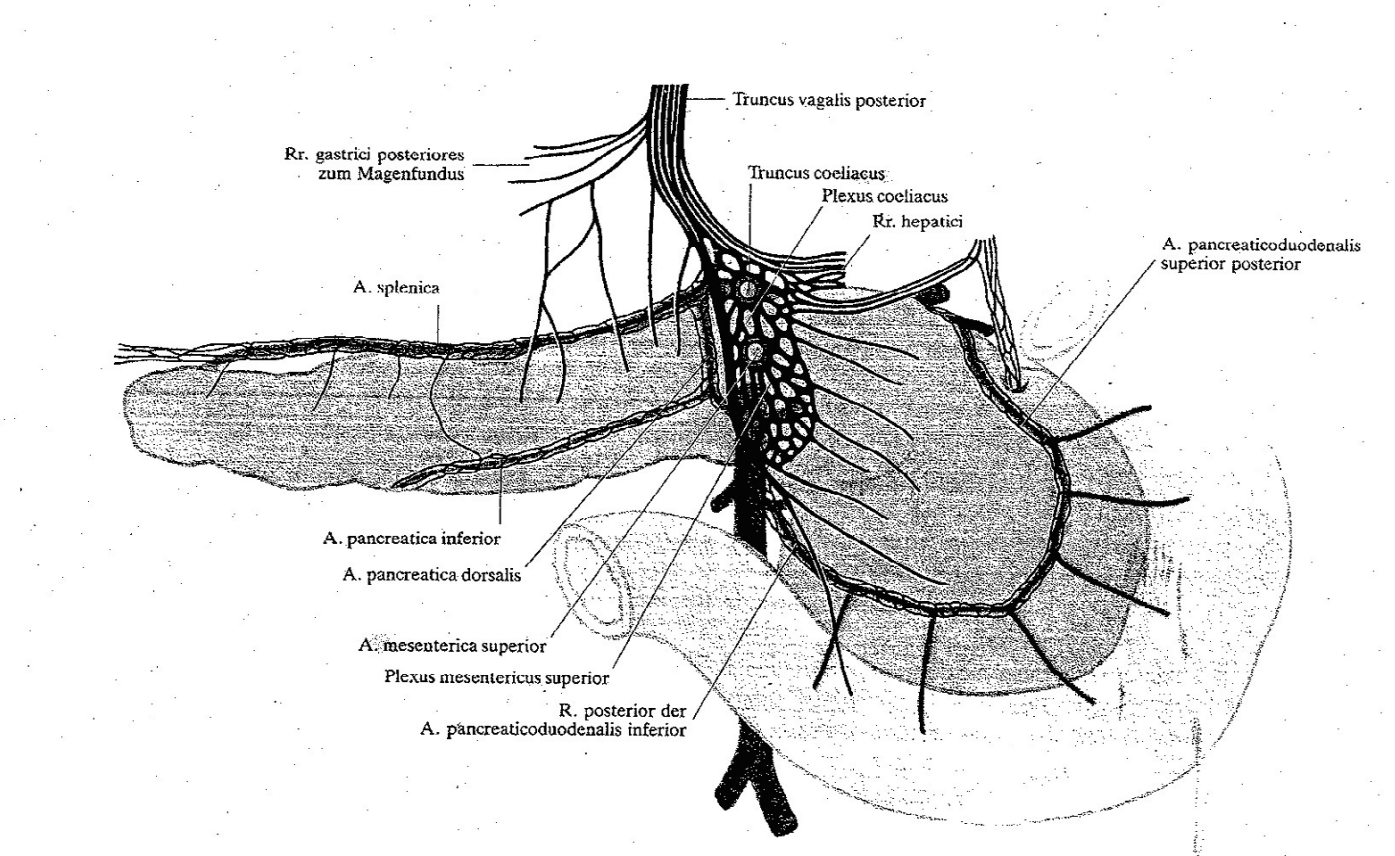
Pancreas with its arteries and nerve plexuses viewed from dorsally. Schematic depiction of pancreatic innervation. Non-vessel bound vegetative nerves and periarterial nerve plexus. (From *Loeweneck *1969, (5)).

Guiding structures in the dissection of the mesopancreas are thus the nerve plexuses dorsal to the pancreas. Of particular importance for the resection of the head of the pancreas are the celiac, hepatic and superior mesenteric plexuses, as well as the choledochus plexus. Figure 1 shows further significant nerve fibers which course periarterially posterior to the hepatic, superior mesenteric, and pancreaticoduodenal arteries [5]. The neural structures on both sides of the superior mesenteric artery – the plexus mesentericus I and II – are components of the Japan Pancreas Society staging system of pancreatic cancer (Classification of pancreatic carcinoma, 1^st ^English Edition, Tanehara *et al*. Tokyo 1996). Recently, the innervation of the pancreatic and retropancreatic tissue has been well studied in the surgical-anatomy literature [6,7].

## Materials and methods

Preparation of the mesopancreas was performed in 5 fresh corpses (2 females, 3 males) ranging from 78 to 84 years of age. All cases had put the body donation at the Institute of Anatomy and Cell Biology of the Johannes Gutenberg-University's disposal after death. The deceased were frozen at a maximum of one day after death and stored between one week and 13 months at -30°C. 36–48 hours before starting the preparation, the process of thawing was initiated.

### Anatomical dissection

**Figure 2:**
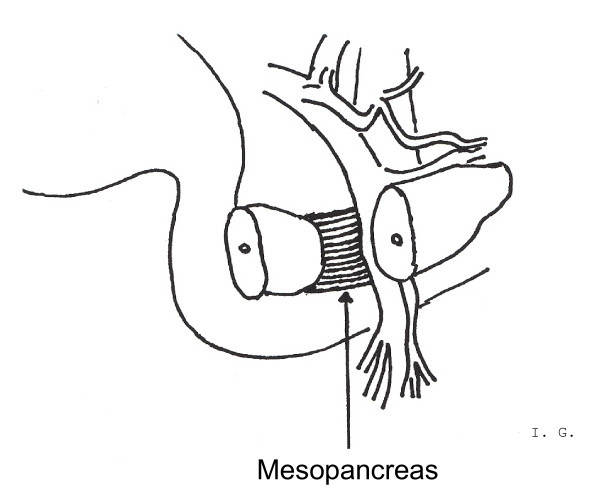
Uncovering the Superior Mesenteric Vein (SMV) and consecutively successive separation of the mesopancreas directly lateral of the Superior Mesenteric Vein (SMV).

After division of the pancreatic neck the mobilized resectate was turned to the right side. On that occasion, the firm and well-vascularized mesopancreas, drawing from the back surface of the pancreatic head, strained itself. Consecutively, the Superior Mesenteric Vein (SMV) was uncovered and afterwards the successive separation of the mesopancreas was performed directly lateral of the Superior Mesenteric Vein (SMV) (Figure 2).

### Histologic and immunohistochemical examinations

**Figure 3:**
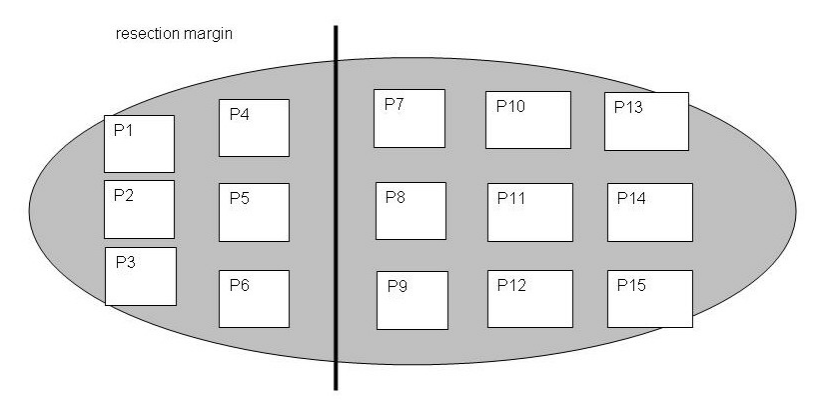
Schematic depiction of the extraction of specimens (aP1-aP15 = specimens 1–15) of the mesopancreas for histologic and immunohistochemical examinations (ventral view after separation from the pancreas).

Pancreas and mesopancreas were separated from each other and the mesopancreas was prepared according to the schematic depiction of the extracted specimens in Figure 3 to 15 blocks (aP1-aP15) for histological and immunohistochemical examinations.

Mirror-wise, the corresponding blocks (bP1-bP15) were taken from the dorsal site of the pancreas. Perivascular nerve fibres were worked up separately (cP1-cP4). The blocks were fixed in 7% formalin and cut to 7 μm-thin layers. Staining was carried out with Haematoxylin and Eosin (H&E) and the monoclonal antibody D2-40 (mouse anti-human) (Dakocytomation) for the selective representation of lymphatic endothelium and ganglion cells.

## Results

### Preparation of the mesopancreas in fresh corpses

**Figure 4:**
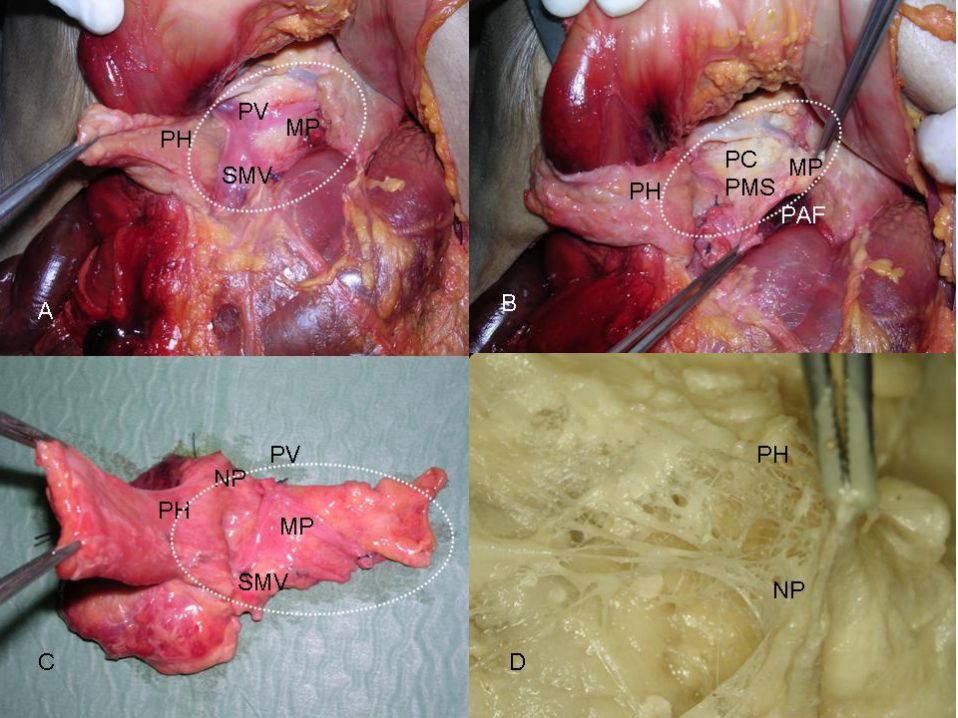
**A)**. The pancreatic head was dissected laterally of the Superior Mesenteric Vein (SMV) and is turned to the right side after mobilization. The mesopancreas strains itself dorsally of themesenteric vessels as a whitish-firm, fatty tissue-like layer. **PH **= Pancreatic Head; **SMV **= Superior Mesenteric Vein; **PV **= Portal Vein; **MP **= Mesopancreas . **B)**. Resection of the pancreas and mesopancreas dorsally of the mesenteric vessels en bloc. **PH **= Pancreatic Head; **MP **= Mesopancreas ; **PAF **= Preaortic Fascia, **PC **= Plexus Coeliacus, **PMS **= Plexus Mesentericus superior. **C)**. Macroscopically, in the dissected en-bloc specimen of the pancreas and mesopancreas nerve plexuses are found running from the dorsal surface of the pancreatic head to the mesopancreas to establish a perineural plane. **PH **= Pancreatic Head;**NP **= Nerve Plexus; **SMV **= Superior Mesenteric Vein; **PV **= Portal Vein; **MP **= Mesopancreas . **D)**. The nerve plexus of the dorsal site of the pancreatic head is depicted in a detailed view after preparation of the layer between pancreas and mesopancreas. **PH **= Pancreatic Head; **NP **= Nerve Plexus of the dorsal margin of the pancreatic head.

In 5 fresh corpses preparation of the mesopancreas was performed. The pancreatic head was dissected laterally of the Superior Mesenteric Vein (SMV) and is turned to the right side (Figure 4a) after mobilization. The mesopancreas strains itself dorsally of the mesenteric vessels as a whitish-firm, fatty tissue-like layer.

The dimensions and extensions of the mesopancreas are defined by the embryology. As soon as the ventral bud rotates and attaches to the posterior body wall, the nerve plexus containing connective tissue layer is positioned dorsally and will attach to the posterior body wall. Right laterally the mesopancreas extends to the descending duodenum, left laterally behind the spleen. Caudally the mesonpancreas may well extend below the mesenteric vessels.

In a next preparation step the resection of the pancreas and mesopancreas dorsally of the mesenteric vessels was done *en bloc *(Figure 4b) (for better presentation a separation of both structures was already performed at the resection margin, the pancreatic head was resected as an intact entity comprising the pancreas and the mesopancreas).

Superior Mesenteric Vein (SMV) and Superior Mesenteric Artery (SMA) are marked by a suture. The mesopancreas is grasped by a tweezers at the lateral resection margin and the preaortic fascia presents itself dorsally.

Macroscopically, in the dissected *en-bloc *specimen of pancreas and mesopancreas nerve plexuses were found running from the dorsal site of the pancreatic head to the mesopancreas to establish a perineural plane (Figure 4c). The resected pancreatic head/-corpus was mobilized and turned to the right side. Portal Vein (PV), Superior Mesenteric Vein (SMV) and Splenic Vein (SV) are marked by a suture.

The nerve plexus of the dorsal surface of the pancreatic head is depicted in a detailed view after preparation of the layer between pancreas and mesopancreas in Figure 4d.

### Histological and immunohistochemical examinations

**Figure 5:**
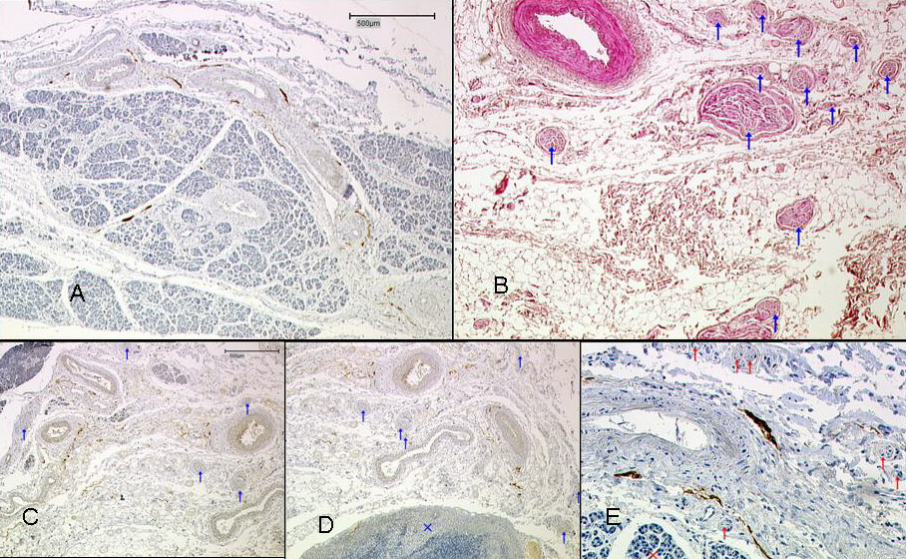
**A)**. Pancreatic tissue and surrounding vessel-nerve-bundles. Lymphatic vessels stained brown (D2-40). B). Nerve fibres embedded in the fatty tissue of the mesopancreas. Nervesare marked with blue arrows (H&E). C) Mesopancreas, pancreatic tissue in the left upper area. Vessels and nerves (blue arrows) embedded in fatty tissue, lymphatic vessels stained brown (D2-40).D). x = Lymph node in the mesopancreas, lymphatic vessels stained brown. E). Mesopancreas; x = pancreatic tissue, red arrows: nerves; lymphatic vessels stained brown.

The histological and immunohistochemical examinations show the lymphatic vessels localized in direct vicinity of the neuronal plexuses between pancreas and mesopancreas (Figure 5a–e).

## Discussion

The 5-year survival rates after partial duodenopancreatectomy cited in the literature range from 7–25%, at a median survival period of 11–20 months for all tumor stages [8-11].

Lymph node metastases, frequently with involvement of several lymph node groups, are found in 20–77% of resection specimens from patients with carcinoma of the head of the pancreas. However, the extent of lymphadenectomy does not exert a significant influence on the prognosis: the results of two randomized studies, one being a multicenter trial [12], did not demonstrate a survival benefit for patients after extended lymphadenectomy [12,13]. Furthermore, neither a prospective, non-randomized study [14] nor a retrospective trial [15] reported an improvement in long-term survival comparing outcomes after extended retroperitoneal lymphadenectomy during pancreaticoduodenectomy for ductal adenocarcinoma of the pancreas with results following the standard procedure.

Nodal micrometastases identified in the resection specimens by immunohistochemistry or molecular diagnostic methods did not have a significant influence on either prognosis or survival [16].

Fortner introduced the principle of radical "regional pancreatectomy" in 1973 [17]. The procedure consists of a total pancreaticoduodenectomy with distal gastrectomy and dissection of the transpancreatic segment of the portal vein. Further resected are the celiac axis, the superior mesenteric artery, and the middle colonic artery with angioplasty [17,18].

Resection of the portal or the superior mesenteric vein is justified to achieve both an adequate safety distance from the tumor and tumor-free resection margins. Various authors did, however, not report a survival benefit after this technique compared with outcomes following a standard procedure [19-21].

The responsibility for the prevention of loco-regional recurrence in patients with pancreatic cancer lies to a high degree with the surgeon. The improvement of long-term survival rates therefore necessitates complete clearance of any extrapancreatic tumor seeding.

**Figure 6:**
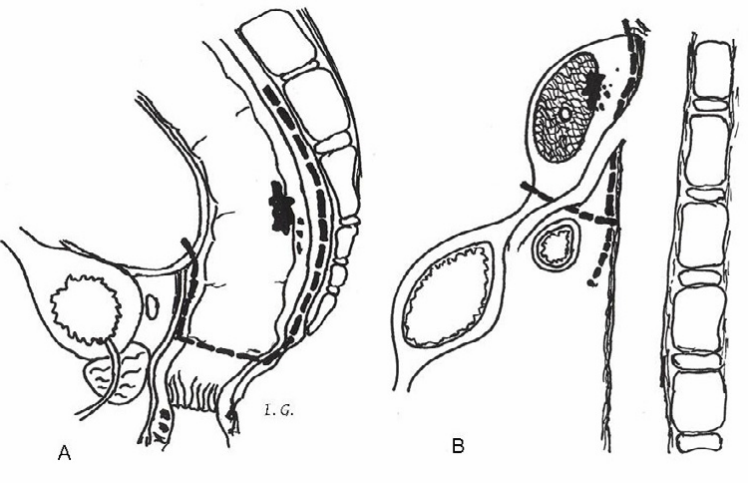
RMP in pancreatic cancer compared to TME in rectal cancer.

The available literature on the surgical anatomy of the pancreas does not contain a single description of the mesopancreas as a perineural lymphatic layer located dorsally to the pancreas. This may be accounted for by the fixation techniques used, which cause adhesion of the pancreatic and mesopancreatic tissue planes in the anatomic specimens and do not permit a macroscopic differentiation between the two layers. Our histological and immunohistochemical investigations showed the lymphatic tracts to course in the immediate vicinity of the neuronal plexus between the pancreas and the mesopancreas. The function of lymphatic structures in the neuronal peripancreatic tissue has previously been well reported in the surgical literature [4,22-26]. Thus, their precise anatomic delimitation or histomorphologic characteristics have not been addressed thus far. Consequently, a PubMed online search did not yield any reference to the term mesopancreas. Our study demonstrates the need for a new surgical classification – the mesopancreas – which represents a known space of oncological relevance: (Postulated in analogy to total mesorectal excision (TME) (Figure 6) is the *en-bloc *resection of the peripancreatic tissue, which optimally consists of the removal of the target organ and the surrounding layer of fat as an intact entity.

It is well established that lymphangiosis carcinomatosa can be detected in the retropancreatic spaces around the nerve plexus in 67% of pancreatic cancer stage I and II [27] and in 88% of cancer stage III and IV [28].

In addition to tumor spread into the extrapancreatic plexus via the lymph vessels and venous canals, the perineural space has been described as an independent route for dorsal cancer infiltration [29]. Nagakawa *et al*. did not establish a correlation between extrapancreatic nerve plexus invasion and lymph node metastasis, although nerve plexus invasion was more pronounced in their patients with extensive lymph node involvement [30]. Conversely, Takahashi *et al*., found a statistically significant difference for the presence of lymph node metastases, but not for the extent of lymph vessel invasion between patients with and without nerve plexus involvement [29]. These findings suggest that tumor spread into the retropancreatic plexus may be due to malignant cell invasion from adjacent loco-regional lymph nodes.

However, the importance of the retropancreatic space invasion in pancreatic cancer is not new and especially the retroperitoneal resection margin and vessel involvement have been emphasized as important factors determining survival after pancreaticoduodenectomy for ductal adenocarcinoma of the pancreas [1].

An important disadvantage of radical lymphadenectomy all around the superior mesenteric artery frequently seen might be the occurrence of severe postoperative diarrhoea.

An exact understanding of the anatomy of the perineural efferent lymphatic vessels is of paramount importance in the surgical-oncological therapy for pancreatic cancer.

The aim to ensure the greatest possible distance from the retropancreatic lymphatic tissue which drains the carcinomatous focus can be achieved in patients with pancreatic cancer only by complete resection of the mesopancreas. The results of this study suggest that the application of radical surgical concepts may be justified to achieve a possible improvement in the long-term outcome after surgical therapy for pancreatic cancer. It becomes apparent that in particular patients with early tumor stages may benefit from the described surgical principle as a result of the associated decrease in loco-regional occurrence [31]. In contrast to local recurrence, the radicality of the surgical procedure is of no influence on the development of distant metastases in patients with pancreatic cancer.

## Conclusion

It has been demonstrated conclusively that patients with larger tumors (T3) and the presence of retroperitoneal invasion are at high risk for the development of distant metastasis [23]. The described concept for resection of the mesopancreas (RMP) needs to be evaluated by future clinical studies to clearly define the target group which will benefit from this form of surgery.

## Conflict of interests

The author(s) declare that they have no competing interests.

## Authors' contributions

**I.G., M.A.K. and T.J**. initiated the presented study, participated in its design and carried out the study. **I.G., M.D., T.W. and M.A.K**. performed the preparations of the mesopancreas. **T.W. and M.A.K**. carried out the histopathological evaluation. **I.G., M.A.K. and T.J**. coordinated of the study. **I.G. and M.D**. drafted the first version of the manuscript and all authors read and approved the final manuscript.
